# A cross-sectional comparative study on the effects of body mass index and exercise/sedentary on serum asprosin in male college students

**DOI:** 10.1371/journal.pone.0265645

**Published:** 2022-04-05

**Authors:** Ruiqi Huang, Chenglin Song, Tao Li, Caijing Yu, Tingting Yao, Haining Gao, Shicheng Cao, Xuejie Yi, Bo Chang

**Affiliations:** 1 Department of Human Kinesiology, Shenyang Sport University, Shenyang, Liaoning, China; 2 Exercise and Health Research Center, Shenyang Sport University, Shenyang, Liaoning, China; The Ohio State University, UNITED STATES

## Abstract

Adipocytes regulate the body’s metabolism by secreting adipokines to maintain energy homeostasis. Asprosin is a new type of adipokine, and its relationship with obesity remains controversial. There are a few reports on the effect of long-term exercise on serum asprosin level. This study aimed to investigate the effects of body mass index (BMI) and exercise/sedentary habit on serum asprosin in male college students as well as the relationship between serum asprosin and body composition and related metabolic indicators and provided a basis for further exploration of the biological function of asprosin. Ninety-six male college students were classified into the sedentary habit group (SD; 48) and the special training experience group (ET; 48). Both groups included three subgroups of normal BMI, overweight, and obesity, with 16 people in each subgroup. One-way analysis of variance, analysis of covariance, and Pearson correlation analysis were performed. The results showed that serum asprosin levels in the obesity subgroup were significantly higher than those in the normal and overweight subgroups. Excluding BMI interference, there were no significant differences in serum asprosin between the SD and ET groups; however, there were significant differences in body composition, tumor necrosis factor-α, interleukin-6, and interleukin-10. Asprosin was positively correlated with BMI, body fat percentage, visceral fat area, fasting insulin, insulin resistance homeostasis model, total cholesterol, low-density lipoprotein, tumor necrosis factor-α, interleukin-6, and leptin levels and was negatively correlated with relative lean body mass, relative skeletal muscle mass, high-density lipoprotein, and interleukin-10, and adiponectin levels. In conclusion, serum asprosin is closely related to body weight, body composition, glucose and lipid metabolism, inflammatory response, and fat hormones. Long-term exercise training cannot prevent BMI increase from increasing serum asprosin level. If the influence of BMI is excluded, long-term exercise training does not affect serum asprosin.

## Introduction

According to the World Health Organization, the incidence of obesity has almost tripled in the past 40 years worldwide [1], and it is estimated that one-third of the world’s population is overweight or obese [2]. Obesity can significantly increase the risk of cardiovascular and cerebrovascular diseases, diabetes, fatty liver, and other related diseases [3] and poses huge challenges to human health. Exploring the mechanism of obesity and identifying effective intervention methods have become the focus of global attention.

Adipose tissue is recognized as one of the largest endocrine organs in the human body. It can secrete many adipokines, including leptin, adiponectin (ADPN), and resistin and inflammatory factors such as tumor necrosis factor (TNF)-α, interleukin (IL)-6, and IL-10. These adipokines have diverse functions and are potential targets for the prevention and treatment of obesity [4–6]. In 2016, Romere et al. [7] discovered a new type of adipose factor, asprosin (ASP), which can not only stimulate hepatocytes to release blood glucose but also cross the blood-brain barrier, activate orexigenic AgRP neurons in the arcuate nucleus of the hypothalamus, and simultaneously inhibit anorexic POMC neurons to stimulate appetite [8]. This suggests that white fat is involved in maintaining the body’s energy balance. Research has shown that subcutaneous injection of recombinant ASP into mice that fasted for 2 hours resulted in an immediate increase in blood glucose levels, leading to compensatory hyperinsulinemia that returned to normal after 1 h. In a mouse mode of insulin resistance, injection of specific monoclonal antibodies resulted in decreased plasma ASP levels, which reduced hepatic glucose release and improved insulin sensitivity [7]. These studies indicate that ASP plays an important role in regulating insulin sensitivity. Previous studies also explored the level of ASP in different body mass index (BMI) populations. Although the majority of previous studies reported a positive correlation between ASP and BMI, the few reported results are diametrically opposite [9–11]. Additionally, more obese children and less obese youths have been studied; therefore, more population-based studies are warranted.

Regular exercise of a certain intensity and load is an extremely good lifestyle intervention that can reduce BMI levels and improve insulin resistance [12–14]. However, few studies have explored the impact of exercise on ASP levels. The available studies mainly focused on the effect of acute exercise, and these have shown inconsistent results, with some studies showing that exercise increases serum ASP level and others showing no change [15–17]. However, there are no reports on the effect of long-term exercise training on serum ASP levels. Therefore, this study conducted a cross-sectional comparative analysis of male college students with different BMIs, who have sedentary habits or special training experience, to explore the effects of BMI and exercise/sedentary habit on serum ASP, as well as the relationship between serum ASP and metabolism-related indicators; accordingly, this study provides an experimental basis for further exploration of the biological mechanism of ASP.

## Materials and methods

### Research subjects

**Fig 1:**
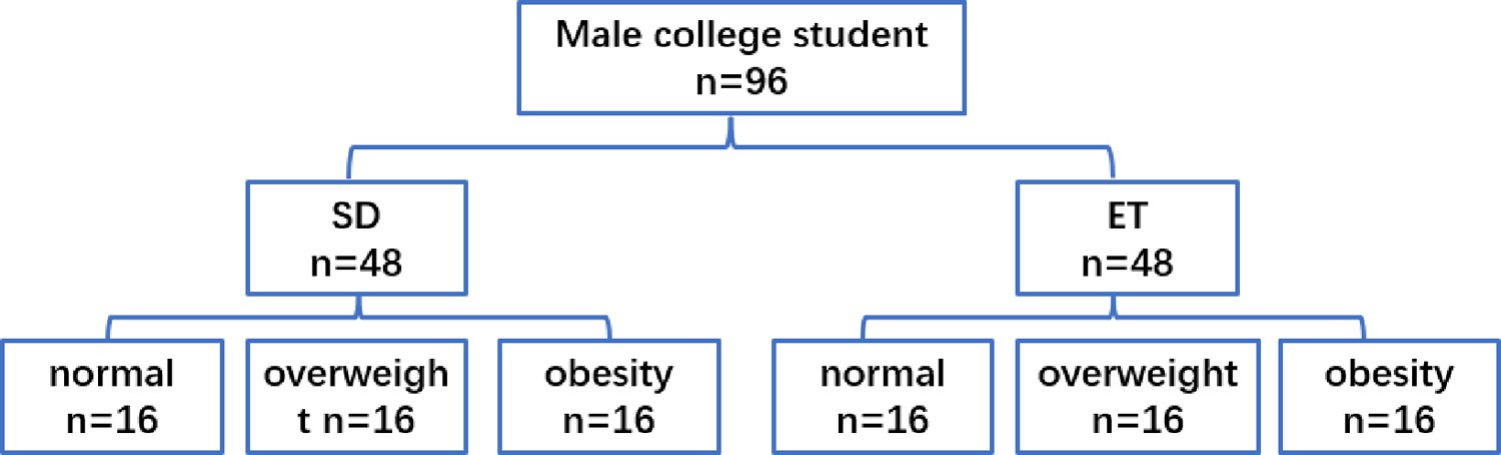
Basic characteristics of all participants.

We recruited ordinary male college students and sports major college student volunteers for physical examinations. Volunteers’ sedentary time, physical activity, and sports training were surveyed through questionnaires. Students with diseases (acute cardiovascular and cerebrovascular diseases, abnormal liver function, and chronic kidney disease) and smoking and alcohol intake were excluded (S1 File). According to the Chinese obesity standard [18], these students were classified into the normal BMI, overweight and obese groups (Fig 1). Ordinary college students did not participate in special sports training; their physical activity frequency was less than three times a week and <0.5 h of low-intensity exercise each time, and sedentary time was 7.7 ± 1.0/day; accordingly, they were included in the sedentary group (SD) [19, 20]. College students majoring in physical education received >5 years of special training and obtained the second-level athlete certificate issued by the Municipal Sports Bureau. Sports training frequency was more than 4 times a week for 1.5–2 h each time, moderate-intensity and above exercise was >1 h, and sedentary time was 4.2±1.1/day; accordingly, they were included in the exercise training group (ET). The study methods were performed in accordance with the Declaration of Helsinki. This study was approved by the ethics committee of the Shenyang Institute of Physical Education. Before study initiation, the study plan was explained to the participants, and written informed consent was obtained from all participants.

### Body composition determination

Using the bioelectrical impedance method [21], the Inbody770 body composition meter was used to measure height, weight, BMI, lean body mass, body fat mass, body fat percentage (PBF), skeletal muscle mass, skeletal muscle index (SMI), and visceral fat area (VFA) and calculate the relative lean body mass (RLBM) and relative skeletal muscle mass (RSMM).

### Blood sample collection

After fasting overnight for 8 h, 5 mL of venous blood was collected from the cubital fossa of the forearm of all participants and centrifuged at 3000 r/min for 15 min. The serum was separated into a sterile Eppendorf tube and sealed at -80°C.

### Biochemical analysis

Fasting blood glucose (FBG) and fasting serum insulin (FINS) levels were assessed using the glucose oxidase method and enzyme-linked immunosorbent assay (ELISA), respectively. Fasting blood glucose kit was purchased from Nanjing Jiancheng Bioengineering Institute (F006-1-1). The insulin kit was purchased from Cloud-Clone Corp., Wuhan, China (CEA448Hu). Insulin resistance homeostasis model (HOMA-IR) level was calculated using the following formula: HOMA-IR = FINS (mIU/L) × FBG (mmol/L) / 22.5 [22].

The GPO-PAP method was used to determine triglyceride (TG) levels, and the COD-PAP method was used to determine total cholesterol (TC) level; a two-reagent direct method was used to determine high-density lipoprotein cholesterol (HDL-C) and low-density lipoprotein cholesterol (LDL-C) levels. The kits were purchased from Nanjing Jiancheng Bioengineering Institute (A110-1-1, A111-1-1, A112-1-1, and A113-1-1, respectively).

### Determination of related adipokine and inflammatory factor levels

ELISA was used to determine the levels of serum ADPN and leptin, and the kits were purchased from Wuhan Yunclone Technology Co., Ltd. (SEA605Hu and SEA084Hu, respectively). ELISA was used to determine the levels of TNF-α, IL-6, and IL-10 (ml077385, ml055488, and ml064299, respectively; mlbio, Shanghai). The specific operation method was strictly performed according to the manufacturer’s instructions.

### Serum ASP level determination

ELISA was used to determine the serum ASP level, and tests were strictly performed according to the manufacturer’s instructions. The kit was purchased from ELAAB Science Inc, Wuhan (E15190h). The range of intra-assay and inter-assay coefficients of variation was between <10% and <12%, respectively, and the determination range of this kit was 3.90–250 ng/mL.

### Statistical analysis

Normal distribution was evaluated using the Kolmogorov–Smirnov test. Data were expressed as the mean±standard deviation. One-way analysis of variance (ANOVA) was used to analyze different BMI groups, and post-hoc comparison analysis was performed; in the comparison of the SD and ET groups, covariance analysis was employed to avoid the interference caused by BMI. Pearson’s correlation analysis was used to analyze the correlation between ASP levels and other parameters. In all statistical analyses, statistical significance was set at P<0.05. SPSS25.0 (IBM, Armonk, NY, USA) was used for statistical analysis.

## Results

### Comparison of related variables according to different BMI grades in the SD and ET groups

In the SD group, one-way ANOVA showed significant differences in SMI and TG among the normal, overweight, and obese subgroups (P<0.05); similarly, significant differences were observed in BMI, RLBM, PBF, RSMM, VFA, FBG, FINS, HOMA-IR, TC, HDL-C, LDL-C, TNF-α, IL-6, IL-10, ADPN, leptin, and ASP levels (P<0.01) (Table 1).

**Table 1:** Comparison of related variables of different BMI grades in SD/ET group.

	SD			ET		
	Normal (n = 16)	Overweight (n = 16)	Obesity (n = 16)	Normal (n = 16)	Overweight (n = 16)	Obesity (n = 16)
BMI (kg/m^2^)	22.01±0.99	25.79±0.94^a^^a^	32.37±3.22^a^^a^^b^^b^	21.58±1.49	25.53±0.98^a^^a^	30.72±1.76^aabb^
RLBM	0.84±0.02	0.78±0.03^a^^a^	0.68±0.05^a^^a^^b^^b^	0.87±0.03	0.81±0.05^a^^a^	0.73±0.04^aabb^
PBF (%)	16.41±2.22	22.42±2.75^aa^	32.15±4.57^a^^a^^b^^b^	13.47±2.82	18.73±4.59^a^^a^	26.69±3.53^aabb^
SMI (kg/m^2^)	9.53±0.90	10.45±1.28^a^	10.94±1.61^a^^a^	10.57±0.79	11.79±0.54^a^^a^	11.85±1.62^aa^
RSMM	0.43±0.04	0.41±0.05	0.34±0.05^a^^a^^b^^b^	0.49±0.02	0.46±0.03^a^^a^	0.39±0.06^aabb^
VFA (cm^2^)	47.81±8.70	68.89±8.86^a^^a^	130.88±38.58^a^^a^^b^^b^	34.24±10.27	61.16±18.03^a^^a^	100.76±19.86^aabb^
FBG(mmol/L)	5.02±0.24	5.26±0.28^a^	5.33±0.29^a^^a^	5.09±0.21	5.23±0.29	5.33±0.48
FINS (mIU/L)	8.53±1.21	10.03±1.09^a^^a^	12.30±1.92^a^^a^^b^^b^	8.87±0.66	10.31±0.56^a^^a^	11.88±0.89^aabb^
HOMA-IR(*10^-6^mol*IU*L^-2^)	1.91±0.34	2.34±0.26^a^^a^	2.92±0.49^a^^a^^b^^b^	2.00±0.13	2.39±0.20^a^^a^	2.81±0.30^aabb^
TC (mmol/L)	4.01±0.76	4.28±0.83	5.40±0.90^a^^a^^b^^b^	3.78±0.70	4.22±0.87	4.56±0.70^aa^
TG (mmol/L)	0.72±0.19	0.75±0.16	1.00±0.40^a^^b^	0.70±0.21	0.74±0.24	0.88±0.32
HDL-C (mmol/L)	1.46±0.15	1.29±0.16^a^^a^	1.14±0.13^a^^a^^b^	1.50±0.13	1.32±0.16^a^^a^	1.17±0.15^aab^
LDL-C (mmol/L)	2.10±0.32	2.70±1.26	3.36±1.09^a^^a^	2.05±0.70	2.51±0.49^a^	2.92±1.04^a^
TNF-α (pg/mL)	17.43±3.75	25.35±4.61^a^^a^	30.16±5.93^a^^a^^b^	16.40±2.70	22.63±3.83^a^^a^	25.51±2.72^aabb^
IL-6 (pg/mL)	5.80±0.68	6.52±0.93^a^	7.00±1.11^a^^a^	5.27±0.37	5.63±0.40^a^	5.97±0.78^aa^
IL-10 (pg/mL)	894.57±69.02	791.14±81.16^a^	715.63±105.37^a^^a^^b^	943.50±67.61	837.01±58.06^a^^a^	777.50±85.68^aab^
ADPN (ng/mL)	2621.33±418.68	2225.50±886.06	1581.33±354.91^a^^a^^b^	2969.14±736.15	2218.01±841.47^a^	1678.01±574.08^aab^
Leptin (ng/mL)	1.43±0.06	1.44±0.06	2.31±0.61^a^^a^^b^^b^	1.35±0.04	1.50±0.16^a^^a^	2.25±0.54^aabb^
ASP (ng/mL)	23.77±11.56	33.11±22.89	82.06±44.99^a^^a^^b^^b^	20.09±11.96	30.49±18.61	92.98±46.39^aabb^

Note: BMI: body mass index; RLBM: relative lean body mass; PBF: body fat percentage; SMI: skeletal muscle index; RSMM: relative skeletal muscle mass; VFA: visceral fat area; FBG: fasting blood glucose; FINS: fasting insulin; HOMA-IR: insulin resistance homeostasis model; TG: triglycerides; TC: total cholesterol; HDL-C: high-density lipoprotein cholesterol; LDL-C: low-density lipoprotein; TNF-α: tumor necrosis factor α; IL-6: interleukin 6; IL-10: interleukin 10; ADPN: adiponectin; ASP: asprosin.

^a^compared with the normal BMI group

^b^compared with the overweight BMI group.

P<0.05 indicates a significant difference; P<0.01 indicates an extremely significant difference.

Post-hoc analysis results showed that compared with the normal BMI group, the levels of BMI, PBF, SMI, VFA, FBG, FINS, HOMA-IR, TNF-α, and IL-6 were significantly increased (P<0.05, P<0.01) and the levels of RLBM, HDL-C, and IL-10 were significantly decreased in the overweight group (P<0.05, (P<0.01). Furthermore, compared with the normal BMI group, the levels of BMI, PBF, SMI, VFA, FBG, FINS, HOMA-IR, TC, TG, LDL-C, TNF-α, IL-6, leptin, and ASP were significantly increased (P<0.05, P<0.01) and those of RLBM, RSMM, HDL-C, IL-10, and ADPN were significantly decreased in the obese group (P<0.05, P<0.01). Compared with the overweight group, the levels of BMI, PBF, SMI, VFA, FINS, HOMA-IR, TC, TG, TNF-α, leptin, and ASP were significantly increased (P<0.05, P<0.01) and those of RLBM, RSMM, HDL-C, IL-10, and ADPN were significantly decreased in the obese group (P<0.05, P<0.01) (Table 1).

In the ET group, one-way ANOVA showed significant differences in LDL-C and TC among participants in the normal, overweight, and obese subgroups (P<0.05), and significant differences were observed in BMI, RLBM, PBF, RSMM, VFA, FINS, HOMA-IR, HDL-C, TNF-α, IL-6, IL-10, ADPN, leptin, and ASP (P<0.01) (Table 1).

Post-hoc analysis results showed that compared with the normal BMI group, the levels of BMI, PBF, SMI, VFA, FINS, HOMA-IR, LDL-C, TNF-α, IL-6, and leptin were significantly increased (P<0.05, P<0.01) and those of RLBM, RSMM, HDL-C, IL-10, and ADPN were significantly decreased in the overweight group (P<0.05, P<0.01). Furthermore, compared with the normal BMI group, the levels of BMI, PBF, SMI, VFA, FINS, HOMA-IR, TC, LDL-C, TNF-α, IL-6, leptin, and ASP were significantly increased (P<0.05, P<0.01) and those of RLBM, RSMM, HDL-C, IL-10, and ADPN were significantly decreased in the obese group (P<0.05, P<0.01). Compared with the overweight BMI group, the levels of BMI, PBF, VFA, FINS, HOMA-IR, TNF-α, leptin, and ASP were significantly increased (P<0.05, P<0.01) and those of RLBM, RSMM, HDL-C, IL-10, and ADPN were significantly decreased in the obese group (P<0.05, P<0.01) (Table 1).

The above results suggest that as long as the BMI reaches the obesity standard in exercise trainers and people living a sedentary lifestyle, increased serum ASP levels, accompanied by changes in body composition, glucose and lipid metabolism disorders, inflammatory responses, and adipokines secretion disorders, will be observed.

### Comparison of differences in related variables between the SD and ET groups

The results in Table 1 show that BMI is an important factor affecting the level of related variables. Therefore, this study used BMI as a covariate to conduct covariance analysis between the SD and ET groups (Table 2). The results showed that the body components, including RLBM, PBF, SMI, RSMM, and VFA (P<0.01), and inflammation-related factors, including TNF-α (P<0.05), IL-6 (P<0.01), and IL-10 (P<0.05), were significantly different between the two groups. However, no significant differences were observed in glucose and lipid metabolism-related indicators, including FBG, FINS, HOMA-IR, TC, TG, HDL-C, and LDL-C, as well as fat hormones, such as ASP, ADPN, and leptin (P>0.05) (Table 2).

**Table 2:** Analysis of covariance comparison of related variables between SD and ET groups.

	SD (n = 48)	ET (n = 48)	*p*
RLBM	0.76±0.07	0.80±0.07^aa^	<0.001
PBF (%)	23.66±7.29	19.63±6.59^aa^	<0.001
SMI (kg/m^2^)	10.31±1.44	11.41±1.25^aa^	<0.001
RSMM	0.39±0.06	0.45±0.06^aa^	<0.001
VFA (cm^2^)	82.53±42.76	65.38±32.30^aa^	0.001
FBG(mmol/L)	5.20±0.30	5.22±0.36	0.666
FINS (mIU/L)	10.29±2.15	10.35±1.43	0.332
HOMA-IR(*10^-6^mol*IU*L^-2^)	2.39±0.56	2.41±0.40	0.363
TC (mmol/L)	4.56±1.03	4.19±0.82	0.084
TG (mmol/L)	0.82±0.30	0.77±0.27	0.524
HDL-C (mmol/L)	1.30±0.20	1.33±0.20	0.692
LDL-C (mmol/L)	2.72±1.11	2.49±0.85	0.424
TNF-α (pg/mL)	24.31±7.15	21.51±4.92^a^	0.032
IL-6 (pg/mL)	6.44±1.06	5.62±0.63^aa^	<0.001
IL-10 (pg/mL)	800.44±114.62	852.67±100.09^a^	0.023
ADPN (ng/mL)	2142.72±738.79	2288.38±898.24	0.641
Leptin (ng/mL)	1.72±0.54	1.70±0.51	0.569
ASP (ng/mL)	46.31±39.75	47.85±44.24	0.377

Note: BMI: body mass index; RLBM: relative lean body mass; BFP: body fat percentage; SMI: skeletal muscle index; RSMM: relative skeletal muscle mass; VFA: visceral fat area; FBG: fasting blood glucose; FINS: fasting insulin; HOMA-IR: insulin resistance homeostasis model; TG: triglycerides; TC: total cholesterol; HDL-C: high-density lipoprotein cholesterol; LDL-C: low-density lipoprotein; TNF-α: tumor necrosis factor α; IL-6: interleukin 6; IL-10: interleukin 10; ADPN: adiponectin; ASP: asprosin.

P<0.05 means significant difference, represented by ^a^; and P<0.01 means extremely significant difference, represented by ^aa^.

Moreover, our results suggest that when BMI interference is excluded, the ET group still showed higher lean body mass and muscle mass, as well as lower body fat rate and inflammatory response, than the SD group; however, the levels of blood glucose, blood lipid, and fat hormones, especially ASP, had no effect.

### Correlation analysis between serum ASP level and other variables

The correlation between ASP and other clinical variables was evaluated using Pearson’s correlation. The results showed that ASP levels positively correlated with BMI, BFP, VFA, FINS, HOMA-IR, TC, LDL-C, TNF-α, IL-6, and leptin levels and negatively correlated with RLBM, RSMM, and HDL-C, IL-10, and ADPN levels (Table 3).

**Table 3:** Correlation between serum ASP levels and other variables.

	All (n = 96)	
	R	p
BMI (kg/m^2^)	0.551^a^^a^	<0.001
RLBM	-0.518^a^^a^	<0.001
BFP (%)	0.520^a^^a^	<0.001
RSMM	-0.398^a^^a^	0.005
VFA (cm^2^)	0.444^a^^a^	0.002
FINS (mIU/L)	0.535^a^^a^	<0.001
HOMA-IR (*10^-6^mol*IU*L^-2^)	0.537^a^^a^	<0.001
TC (mmol/L)	0.294^a^	0.043
HDL-C (mmol/L)	-0.481^a^^a^	<0.001
LDL-C (mmol/L)	0.574^a^^a^	<0.001
TNF-α (pg/mL)	0.410^a^^a^	<0.001
IL-6 (pg/mL)	0.455^a^^a^	0.001
IL-10 (pg/mL)	-0.280^a^^a^	0.006
ADPN (ng/mL)	-0.457^a^^a^	<0.001
Leptin (ng/mL)	0.417^a^^a^	<0.001

Note: ^a^ indicates the degree of correlation; P<0.05 indicates that the correlation between variables is reliable.

## Discussion

This study shows that serum ASP level increases with an increase in BMI in both trained and sedentary participants, and the serum ASP level of obese participants is significantly higher than that of those with normal BMI, suggesting that exercise training cannot prevent the significant increase in serum ASP caused by obesity. Using BMI as a covariate to perform covariance analysis in the SD and ET groups, we observed significant differences in body composition and serum inflammatory factors between the two groups. However, there was no significant difference in fat hormone level, including ASP. Correlation analysis showed that serum ASP level positively correlated with BMI, BFP, VFA, and FINS, HOMA-IR, TC, LDL-C, TNF-α, IL-6, and leptin levels and negatively correlated with RLBM, RSMM, and HDL-C, IL-10, and ADPN levels.

Asprosin is encoded by the fibrillin-1 (*FBN1*) gene. The RNAseq data provided by Romere et al. [7] showed that *FBN1* mRNA is also expressed in the lung and heart, indicating that adipose tissue is not the only organ expressing *FBN1* mRNA, and circulating ASP levels may be released from multiple organ systems rather than just the adipose tissue. There are still controversies about serum ASP levels in people with obesity, and Long et al. [11] found that compared with children with normal weight, children with obesity had low ASP levels. This is not only inconsistent with the conclusions of two pediatric studies by Sunnetci et al. [23] and Wang et al. [10], but also contrary to the previous conclusions in studies on adult obesity [9, 24] and mouse model studies [7]. This study showed that ASP level increases with increasing BMI. The ASP level in the obesity group was significantly higher than that in the normal BMI and overweight group; however, there was no difference between the overweight and normal BMI groups. Our conclusions further confirm that obesity increases serum ASP level.

Studies have shown that regular physical exercise reduces body fat, promotes metabolism, and improves insulin sensitivity [25, 26]. However, there are few reports on the effect of exercise on serum ASP level, and only a few studies have explored the effect of acute exercise on serum ASP level. The study found that when adult men with normal BMI and overweight/obesity performed 30-min aerobic exercise (intensity of 55–59% heart rate reserve) in the morning and evening, serum ASP levels significantly decreased, and the overweight/obesity group had a greater decline [16]. On the contrary, another study showed that immediately after individuals with obesity undergo acute incremental anaerobic exercise (the heart rate reaches 85% of its maximum value and exercise stops), the serum ASP level in the obesity and normal BMI groups has no significant effect [17]. In addition, 15, 30, and 60 min and 24 h after acute anaerobic exercise (a single 20-s bicycle sprint) in adult healthy men and women, serum ASP level in women significantly changed, whereas men had no significant changes [15]. However, the effect of long-term exercise on human serum ASP levels has not been reported. Existing animal experiments have shown that 8-week continuous swimming exercise (loading body weight of 0–3%) and intermittent swimming exercise (loading body weight 5–16%) can significantly reduce the serum ASP level in Wistar rats with metabolic syndrome [27]. Eight weeks of aerobic treadmill exercise can reduce the expression of ASP in the liver of type 1 diabetes SD rats [28]. Long-term regular exercise can effectively reduce BMI, which is closely related to ASP [9]. To explore the effect of simple long-term exercise training on ASP level, it is necessary to exclude the possible interference of BMI on ASP and other indicators. Therefore, this study used BMI as a covariate when comparing the SD and ET groups. Interestingly, no difference was observed in ASP level between the SD and ET groups, suggesting that long-term exercise training may not affect the serum ASP level.

In this study, there were significant differences in lean body mass, body fat percentage, VFA, and SMI between the SD and ET groups. Correlation analysis showed that serum ASP level positively correlated with BFP and VFA and negatively correlated with RLBM and RSMM. Although ASP is a cytokine secreted by adipose cells, it is also expressed in tissues such as skeletal muscle, lung, and heart. Obesity leads to excessive accumulation of lipids, causing increased expression and secretion of ASP [23, 24], which may be responsible for the positive correlation between obesity-related indicators and ASP. However, why ASP is negatively correlated with RLBM and RSMM remains unclear, and further research is needed.

In addition, this study showed that the ET group had significantly lower levels of inflammatory cytokines (TNF-α and IL-6) than the SD group (with BMI as a covariate). On the contrary, the level of the serum anti-inflammatory factor “IL-10” significantly increased. This further suggests that long-term exercise has a good anti-inflammatory effect. Concurrently, these inflammatory factors were positively or negatively correlated with ASP. Several recent studies demonstrated that the levels of inflammatory factors, such as TNF-α and IL-6, in patients with obesity and type 2 diabetes mellitus, are significantly higher than those in the control group and positively correlated with serum ASP [10, 29, 30]. This is consistent with the conclusions of this study. Furthermore, ex vivo experiments demonstrated that recombinant ASP treatment enhanced the TLR4/JNK-mediated pathway, leading to cellular inflammation and dysfunction. Interestingly, these changes can reduce the effect of ASP on inflammation and cell dysfunction through TLR4 or JNK siRNA [31]. These findings reveal a close relationship between ASP and inflammation, and there were no significant changes in the adipokines ASP, ADPN, and leptin between the ET and SD groups. In the correlation analysis, serum ASP level was found to be positively correlated with HOMA-IR, TC, LDL-C, and leptin levels and negatively correlated with HDL-C and ADPN levels. This is more consistent with the results of other studies [10, 29]. So far, numerous studies have shown a positive correlation between ASP and HOMA-IR [10, 32, 33], and some studies have shown that HOMA-IR may independently affect ASP secretion. The above results indicate that ASP may be a new marker for predicting insulin resistance or type 2 diabetes.

In conclusion, serum ASP is closely related to body weight, body composition, glucose and lipid metabolism, inflammatory response, and fat hormones. Long-term exercise training cannot prevent BMI increase from increasing the serum ASP level. If the influence of BMI is excluded, although long-term exercise training can effectively improve body composition and inhibit the inflammatory response, it does not affect serum ASP levels. It is speculated that although ASP is an adipokine, it can be produced and secreted by multiple organs. Furthermore, ASP is more sensitive to obesity (fat accumulation), and it is unclear whether it may enable the body to adapt to long-term exercise or facilitate an increase in lean body mass and other factors through exercise; therefore, further studies are needed to clarify this phenomenon. Since this is a cross-sectional comparative study of college students involved in exercise training and sedentary habit, it is not possible to understand the continuous changes in serum caused by long-term exercise. Concurrently, the sample size is limited, which is also a study limitation. Therefore, longitudinal and interventional studies assessing the effect of exercise training on serum ASP are required.

## Supporting information

S1 File (XLSX)
